# Gut Microbiota in Anxiety and Depression: Unveiling the Relationships and Management Options

**DOI:** 10.3390/ph16040565

**Published:** 2023-04-09

**Authors:** Akash Kumar, Jhilam Pramanik, Nandani Goyal, Dimple Chauhan, Bhagavathi Sundaram Sivamaruthi, Bhupendra G. Prajapati, Chaiyavat Chaiyasut

**Affiliations:** 1 Department of Food Technology, SRM University, Sonipat 131029, India; 2 Department of Food Technology, ITM University, Gwalior 474001, India; 3 Department of Skill Agriculture, Shri Vishwakarma Skill University, Gurugram 122003, India; 4 School of Bio-Engineering and Food Technology, Shoolini University, Solan 173229, India; 5 Office of Research Administration, Chiang Mai University, Chiang Mai 50200, Thailand; sivamaruthi.b@cmu.ac.th; 6 Innovation Center for Holistic Health, Nutraceuticals, and Cosmeceuticals, Faculty of Pharmacy, Chiang Mai University, Chiang Mai 50200, Thailand; 7 Shree S. K. Patel College of Pharmaceutical Education and Research, Ganpat University, Mehsana 384012, India

**Keywords:** gut–brain axis, microbiota, anxiety, depression, probiotics

## Abstract

The gut microbiota is critical for maintaining human health and the immunological system. Several neuroscientific studies have shown the significance of microbiota in developing brain systems. The gut microbiota and the brain are interconnected in a bidirectional relationship, as research on the microbiome–gut–brain axis shows. Significant evidence links anxiety and depression disorders to the community of microbes that live in the gastrointestinal system. Modified diet, fish and omega-3 fatty acid intake, macro- and micro-nutrient intake, prebiotics, probiotics, synbiotics, postbiotics, fecal microbiota transplantation, and 5-HTP regulation may all be utilized to alter the gut microbiota as a treatment approach. There are few preclinical and clinical research studies on the effectiveness and reliability of various therapeutic approaches for depression and anxiety. This article highlights relevant research on the association of gut microbiota with depression and anxiety and the different therapeutic possibilities of gut microbiota modification.

## 1. Introduction

Depression and anxiety impact people’s everyday life, health, and economic position. According to research, depression may overtake heart failure as the most prevalent disease in the world by 2030 [1,2]. Anxiety and stress are often present in conjunction with depressive disorders; these coexist in approximately 75% of children and adolescents [2,3]. Anxiety disorders are thought to be 47–58% more likely to develop during a depressive episode, and 56% of people with anxiety disorders experience depression [4,5]. Patients’ unexpectedly unpleasant life situations occur around 50% of the time before depressive episodes [6,7,8,9,10]. Unfortunately, everyone experiences stress at some point in their life. With lifetime incidence rates of 14% and 12%, respectively, anxiety and depressive disorders are common mental health conditions globally [11]. It is undoubtedly challenging for the patient to operate in society when suffering from anxiety or despair. The common risk factors for depression are poverty, unemployment, traumatic life events, physical disease, and drug and alcohol addiction, although anybody may suffer from depression. The individual who suffers from anxiety or depression loses their sense of self-worth. In psychiatry, patient observation and medical interviews with the patient and their immediate family members are the primary methods for diagnosing stress, anxiety, and depression [12,13]. In addition to the adverse effects on the individuals affected, these disorders place a significant financial strain on society due to high healthcare costs [14,15]. These points make it evident that there is a need for effective treatments.

Pharmacotherapy is the keystone of current therapies for depression. Selective serotonin reuptake inhibitors (SSRIs) are the most popular first-line medication, although monoamine oxidase and serotonin-norepinephrine reuptake inhibitors are also used. However, the effectiveness of currently available antidepressant medications used in clinics for symptom relief and prevention seems inconsistent [16]. In addition, it has been shown that tolerance develops during follow-up care; using the same medication on the same patient repeatedly leads to decreased efficacy. Up to 35% of people are estimated to experience treatment-resistant depression [17]. Therefore, there is a need to explore novel therapies for preserving the quality of life for all people suffering from depression.

According to studies, there is a correlation between diet, nutrition and anxiety and depression. Preliminary studies suggested that dietary changes may be an alternate treatment or preventative measure for anxiety and depression [18,19]. The correlation between unhealthy diet and the propensity to develop mental illnesses has received more attention in recent years [20]. “Western” dietary patterns with low consumption of fruits and vegetables and high consumption of refined grains, fried and processed meals, red meat, and high-fat dairy products are linked to anxiety and depression [21,22]. At the same time, many correlative studies in healthy adults demonstrate a lower incidence of depression in those who adhere to “healthy” dietary patterns, such as the Norwegian diet [18], Japanese diet [23,24], and Mediterranean diet [25,26], which are focused on the abundant consumption of vegetables, fruits, cereals, nuts, seeds, pulses, dairy, eggs, fish, and unsaturated fats [27]. Stress and depression may also affect dietary preferences, how sweet and fatty foods are perceived [28,29], and taste thresholds [30]. A 10-year longitudinal research study conducted in France demonstrated a correlation between poor nutrition and depression incidence and healthy diet pattern associated with lower depressive symptoms [31]. Randomized controlled trials and prospective research have both shown inconsistent findings when attempting to establish the direction of the correlation. A high-quality diet, independent of its form, with greater fish and vegetable consumption, was related to a decreased incidence of depression, with a dose-type association with compliance with the healthy diet, according to a meta-analysis of prospective studies. In contrast, the meta-analysis revealed that a poor diet was not linked to an increased risk of depression, and the findings revealed significant variability across trials [32].

The scientific findings are not consistent regarding diet and mental health. Maybe there are bidirectional processes behind how the diet might impact anxiety and depression. The micronutrients, such as zinc, magnesium, selenium, iron, and the vitamins B-6, B-12, D, E, and folate, are deficient in those who have depression or are at a greater risk of developing depression and anxiety [33,34,35].

More than 3.8 × 10^13^ bacteria exist in the human gut microbiota [36]. Microflora dysbiosis is associated with increased intestinal permeability and systemic inflammation [37]. The human gut has the second-highest concentration of neurons after the brain [38].

Therefore, studies have been conducted to find the association between gut microbiota and depression. Naseribafrouci et al. confirmed the correlations between this mental disorder and intestinal flora. They showed that individuals with major depressive disorder have higher levels of the genera *Oscillibacter* and *Alistipes* [39]. Lower propionic acid levels and enormous amounts of isocaproic acid were found in individuals diagnosed with depression [40].

Although the etiology of depression is complicated, the gut microbiome’s potential to affect depression development has been examined in several research studies. Intestinal microbiota disturbances might result in the onset of anxiety and depression [41]. After injecting high sugar, high fat, and antibiotic doses into mice, David et al. discovered changes in the mice’s behavior and intestinal microbiota [42]. The quantity of *Bacteroides* spp. dramatically dropped, while *Clostridium* spp. numbers significantly increased [43]. The c-FOS proto-oncogene may be produced by *Campylobacter jejuni*, which can also cause anxiety and depression [44]. *Lactobacillus* and *Bifidobacterium* species were found to be capable of reducing depression to a large extent [45,46]. The points mentioned above suggest that gut microbiota has a role in developing anxiety and depression. Anxiety and depression are gut–brain axis disorders that can be treated with the help of the gut microbiota, which offers a unique approach to modifying neurotransmitter regulation in the brain [47,48].

Studies have shown links between anxiety and depression and the development or diagnosis of certain metabolic disorders [49], cardiovascular diseases [49], cancers [50], atopic illnesses [51], and chronic pain syndromes [52]. Numerous studies on humans and animals have shown the importance of physiological mechanisms underlying inflammatory and stress responses in the etiology of depression and anxiety [53,54]. Extensive research has shown the connection between inflammation and depression [55]. Consumption of a high-fat diet leads to chronic systemic inflammation [56]. Anti-inflammatory medications are beneficial in treating depression [57]. They may change neurotransmitters’ metabolism by lowering precursors’ availability and stimulating the hypothalamic–pituitary–adrenal (HPA) axis. Studies have shown that certain depressed individuals have higher levels of pro-inflammatory cytokines than healthy controls [58]. High levels of pro-inflammatory cytokines indicate a future risk of depression [59]. Studies of individuals with clinically diagnosed anxiety have shown higher levels of pro-inflammatory cytokines than healthy people [58]. Furthermore, it has been shown that triggering an immunological response makes humans [60] anxious and shows anxiety-like phenotypes in animals [61]. On a molecular level, it has been shown that stress-induced interleukin (IL)-6 activity changes the expression of certain genes in monocytes and results in anxiety-like behavior in mice [62]. Though further investigations are required, observational and experimental studies generally provide evidence for the theory that inflammation plays a role in anxiety and depression. This article emphasizes the relationship between gut microbiota and anxiety or depression. We provide a potential strategy for alleviating anxiety and depression that involves modified diets, fish, and omega-3 fatty acids (FA) intake, probiotics, prebiotics, synbiotics, postbiotics, fecal microbiota transplantation, and 5-hydroxytryptophan regulation. The main objective of this article is to summarize the recent evidence linking gut microbiota to anxiety and depression and possible ways to alleviate the symptoms of these disorders.

## 2. Material and Methods

**Figure 1:**
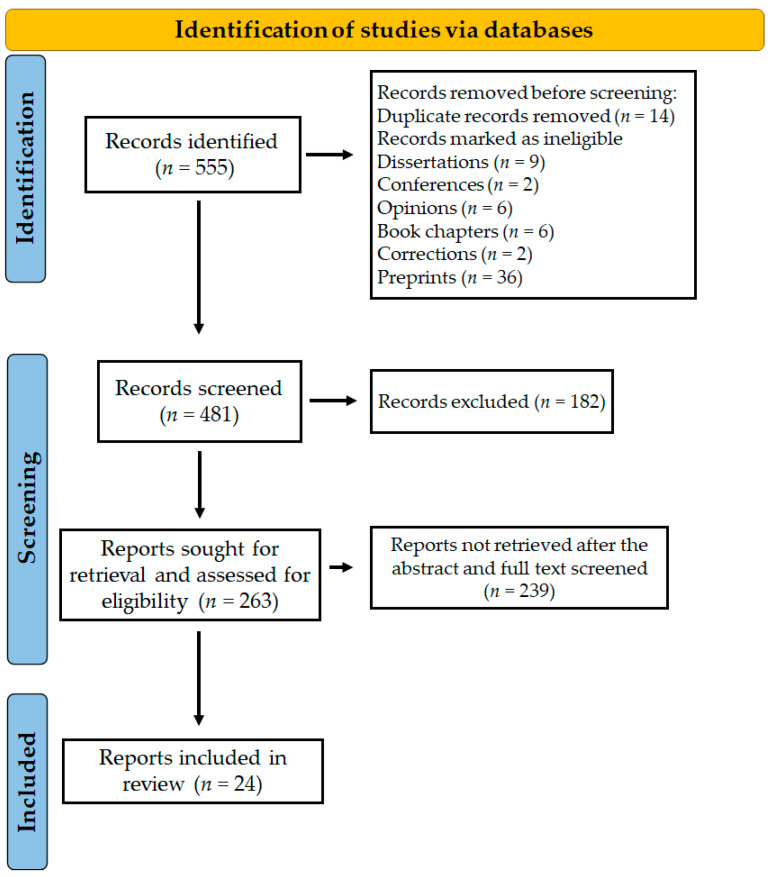
PRISMA diagram explaining the screening and selection of the literature for the study.

Whether there is a link between the gastrointestinal microbiota and anxiety or depression was the major concern of this review. Does the gut microbiota impact the onset, progression, and treatment of anxiety or depression? From 1 November to 31 December 2022, the databases Scopus, Google Scholar, and PubMed were examined using the phrases title:(microbiome OR microbiota) AND title:(depression OR depressive OR anxiety). We provide a thorough summary of the information published on this subject. Figure 1 represents preclinical and clinical data on the relationships of depression and anxiety with alterations in the gut microbiota. This review included 24 papers, most published between 2019 and 2022, indicating a dramatic rise in interest in this field.

## 3. Epidemiology of Depression and Anxiety

### 3.1. The Gut–Brain Axis in Depression and Anxiety

Several studies showed that the gut–brain axis influences the development of anxiety and depression. The gut–brain axis is a network that transmits information in a bidirectional pattern between the gut and the brain and is controlled by neuroendocrine and neuroimmune mechanisms [63,64,65]. Gamma-aminobutyric acid (GABA) neurotransmitters [66], secondary bile acids [67], short-chain fatty acids [68], and tryptophan metabolites generated from the microbiota are only a few of the molecules that regulate these mechanisms [66,69,70]. The gut–brain axis is dysregulated and linked to neuroinflammation and altered blood–brain barrier (BBB) permeability during gut microbiota dysbiosis or disturbance in the gut ecosystem [71]. According to research using rodent models, the BBB becomes more permeable when the normal intestinal microbiota is lost or disturbed [72]. Increased BBB permeability and possible subsequent development of Alzheimer’s disease with amyloid-peptide accumulation may be associated with metabolic illnesses [73]. It has been discovered that microbial dysbiosis affects the protective properties of the BBB, including permeability modulation [72] via tight junction expression [74], and causes behavioral alterations [75].

Studies showed that changes in gut microbiota increased the level of harmful compounds such as *p*-cresol, which may compromise the integrity of the BBB [76,77]. Earlier research demonstrated that *p*-cresol was considerably higher in the prefrontal cortex of susceptible mice that previously exhibited anxiety-like phenotypes [78]. Moreover, the gut-derived metabolite 4-ethyl phenyl sulfate (4EPS) affects brain activity and causes anxiety-like behaviors [79]. The gut microbiota generates reduced levels of neurotoxic metabolites after administration of *Bacteroides fragilis*, including 4-EPS, serum glycolate, and imidazole propionate, improving gut permeability and reducing anxiety-like behavior [80]. Serotonin and dopamine release, brain-derived neurotrophic factor levels, the HPA axis, and the production of inflammatory cytokines may all be affected by disturbances in the gut microbiota during depression and anxiety [81]. For instance, C-reactive protein (CRP) and cytokines, including interleukin-1, interleukin-2, interleukin 6, interleukin-1β, and interferon-γ, were released in response to depression [82].

According to studies, patients with inflammatory disorders are more likely to experience depression. Episodes of depressive symptoms are expected with severe inflammatory bowel disease conditions, possibly related to disrupting the pathways involved in the gut–brain axis [83]. Persistent neuroinflammation alters brain functioning and affects a person’s mood and behavior [56]. The cure for inflammation-mediated depression and vice versa has not been found yet, and managing mental disorders and severe inflammation is challenging [84,85]. People with depression who resist medical treatment might have severe inflammation and gut dysbiosis [86,87].

The research by Guida et al. revealed that the consumption of the probiotic *Lactobacillus casei* was able to alleviate the depression and overall inflammatory state that was caused by antibiotic-induced dysbiosis in mice [88]. It has also been shown that feces from people with depression may cause a depressive-like phenotype in animals with altered gut microbiota [89]. Preliminary evidence from observational studies has demonstrated that patients with depression and anxiety disorders have significantly different gut microbiome profiles compared to healthy individuals [90].

### 3.2. The Relationships between Epigenetics, Gut Microbiota, Depression, and Anxiety

Microorganisms inhabit the human gut in a synbiotic manner. The prokaryotic organisms that comprise the “human holobiont” are essential for preserving homeostasis and proper functioning. Various neurological illnesses, including Alzheimer’s disease, Parkinson’s disease, depression, etc., have been related to disturbances in the gut microbiota composition. Numerous sophisticated molecular mechanisms, including immune system modification [91], metabolic signaling [92], neuroendocrine signaling [93], vagal nerve signaling [94], and epigenetics, are used by these microorganisms to maintain normal homeostasis [95,96]. Epigenetics plays a significant role in controlling host physiology by modifying the metabolic activity of the gut microbiome, which is influenced by environment and nutrition. For example, cofactors for the activity of enzyme acetylases and methylases, which control histone modification and DNA methylation, originate from the gut microbiome. The metabolites generated by the gut microbiota function as cofactors and substrates for numerous enzyme activities [95]. Epigenetic regulation is a dynamic process affected by changes in diet, activity, and microbiota composition [97]. Epigenetics means “in addition to genetics”. Instead of looking at the DNA sequence, it includes analyzing chromosome-level changes in gene expression. Both modifications are persistent and heritable. Epigenetics primarily control chromosomal superstructure changes and chemical modifications to nitrogenous bases without directly affecting the DNA sequence. Epigenetics may result from several molecular processes, but the primary ones include histone modification, DNA methylation and acetylation, and RNA-associated silencing [98].

## 4. Risk Factors Associated with Depression and Anxiety

There are various risk factors for the onset of depression and anxiety, such as biological [99], genetic [100], personality trait-related [101], social [102], economic [103], and lifestyle-related factors [99]. Factors that may contribute to depressive symptoms in the elderly include aging, living alone, being a woman, having less education, getting divorced, having comorbid physical illnesses, having functional disorders, using tobacco and alcohol, and having lower-level cognitive dysfunction [104]. Another meta-analysis found that having a chronic disease and feeling unwell increased the chance of depression [105]. There is a high prevalence of depression in people with physical and chronic illnesses [106]. Several research studies have investigated risk factors for depression and anxiety in students. These factors include age [107], grade [108], ethnicity [109], being an only child [110], attitude toward future career [111], academic pressure [112], smoking addiction [113], alcoholism [113], family financial status [111,112], and social support [114].

## 5. The Gut Microbiota, Depression, and Anxiety

### 5.1. Animal Studies

Numerous studies have looked at the relationship between alterations in the composition and diversity of the gut microbiome and anxiety [115,116,117] and depression [116,118,119]. Most of these studies used rodent models and antibiotics to diminish the gut microbiota [120,121], and the results demonstrated varied behavioral and physiological changes. It is well-acknowledged that stress contributes to the pathophysiology of depression and shows adaptive adjustments in many pathways, including brain-derived neurotrophic factor (BDNF), inflammatory cytokines, and the spleen, to promote resilience [122,123,124]. Learned helplessness susceptible rats had much lower levels of acetic and propionic acid in their feces than the control. The learned helplessness resilient rats had significantly larger relative abundances of the genera *Lactobacillus*, *Clostridium* cluster III, and *Anaerofustis* [125]. Interestingly, resistance to chronic social defeat stress in mice was correlated with antibiotic-induced gut dysbiosis [126]. These results confirm that the brain–gut–microbiota axis influences stress resiliency and vulnerability [123,127]. Preclinical research is accumulating evidence that the brain–gut–microbiota axis is critical in the development of depression [128,129]. Stress-induced depression in rodents has been shown to be accompanied by abnormal levels of gut microbiota-related short-chain fatty acids and other metabolites such as alanine, isoleucine, L-threonine, serine, and tyrosine, which may be connected to altered levels of 5-hydroxytryptamine (5-HT) in the brain and depressive-like phenotypes [130,131]. The brain–gut–microbiota axis is thought to have a bidirectional impact on depression.

The brain, the gut microbiota, and the immune system are connected through the vagus nerve [132]. Vagus nerve signaling, connected to inflammatory control and modification by neuroactive chemicals, is linked to depression [133]. According to Bravo et al., vagotomy prevented the effects of *Lactobacillus rhamnosus* on brain chemistry and depressive-like phenotypes and decreased stress-induced depression-like phenotypes in mice [92]. Subdiaphragmatic vagotomy (SDV) was shown to reduce depression-like phenotypes, levels of pro-inflammatory cytokines, expression of synaptic proteins, and aberrant gut microbiota composition in mice following lipopolysaccharide (LPS) treatment [134]. Researchers have used mouse models of depression to examine how the probiotic *Lactobacillus rhamnosus* affects neural function. One investigation discovered a dependent or independent relationship between variations in the c-Fos protein in certain brain areas and vagal signaling [135]. Considering these results, pinpointing the vagus nerve’s exact pathway to the brain–gut–microbiota axis in depression is of tremendous interest. A stimulating electrode emitting low-frequency, irregular electrical pulses [136] or gut bacteria [137] may stimulate the vagus nerve. Vagus nerve stimulation (VNS) has potent anti-inflammatory actions [138,139]. Patients’ moods improved because of vagus nerve stimulation, first used to treat refractory epilepsy. It is now recognized as a treatment option for those with refractory depression [140].

According to several research studies, it has been proved that the administration of microbiota or their metabolites may exacerbate or ameliorate depression. Fecal microbiota transplantation of the “depression-related microbiome” induced depression-like phenotypes in mice [141,142]. On the other hand, it has been shown that therapy with a combination of short-chain fatty acids, including acetate, butyrate, and propionate, reduces stress-induced depressive behaviors [143]. The altered protein profiles were anticipated to perform roles in the inflammatory immune response and metabolic regulation [144]. The case for the brain–gut axis’ involvement in depression may be strengthened by a better knowledge of the significant changes that occur from the gut to the brain or from the brain to the gut [145,146,147]. In recent research, rats who received feces from depressed human patients displayed depressed behavior and were depleted of *Coproccocus*, indicating that *Coproccocus* may have a causative relationship with depression [141]. To examine and evaluate the effects of microbiota depletion on anxiety-like behavior and depression in rodent models, as well as to identify the research that needs to be performed to assist future translatability, gathering and summarizing the available data is required. Table 1 represents the alteration in microbial composition during anxiety and depression.

### 5.2. Human Studies

The pathogenic effect of gut dysbiosis is linked, in many clinical investigations, to depressive and anxious behaviors [168,169]. Patients with depression typically have disturbed gastrointestinal (GI) symptoms such as constipation, abdominal discomfort, vomiting, nausea, and bloating [170]. Irritable bowel syndrome (IBS) symptoms or psychological distress are often observed in anxious patients [171,172,173]. *Bacteroides*, *Prevotella/Prevotellaceae*, and *Proteobacteria* were more prevalent in comorbid IBS and anxiety/depression patients than in healthy individuals [174]. According to earlier literature, the gut bacterial strains *Coprococcus*, *Subdoligranulum*, *Eggerthella*, and *Ruminococcaceae* are linked to depression [175]. It was consistently shown that *Eggerthella* levels were higher in those with depression and anxiety. It was shown that those with depression and generalized anxiety disorder had decreased levels of *Subdoligranulum* and *Coprococcus* [148]. Both unipolar and bipolar depression patients have been discovered to have low levels of the genus and family *Ruminococcaceae* [148,149,176]. Bosch et al. study also showed a similar trend, with numerous taxa from the family *Ruminococcaceae* being decreased among individuals reporting more depressive symptoms. Depression symptoms were strongly correlated with *Sellimonas* and *Hungatella*, and higher levels of *Lachnoclostridium* were linked to greater depression symptoms [175].

Some of the key points must be considered in microbiome studies. In detail, genetic variation affects the gut microbial composition and diversity and disease incidences in humans [177]. The study revealed that despite differences in microbial composition between humans and mice, the results of animal studies aid in improving the understanding of the interplay between host genetics and gut microbiota [178]. We must consider the study population’s genetic makeup, age, traditions, and geography when we study their microbial composition. Additionally, lifestyle and food habits affect microbial compositional variation [179].

## 6. Potential Therapy Involved in the Treatment of Depression and Anxiety

**Figure 2:**
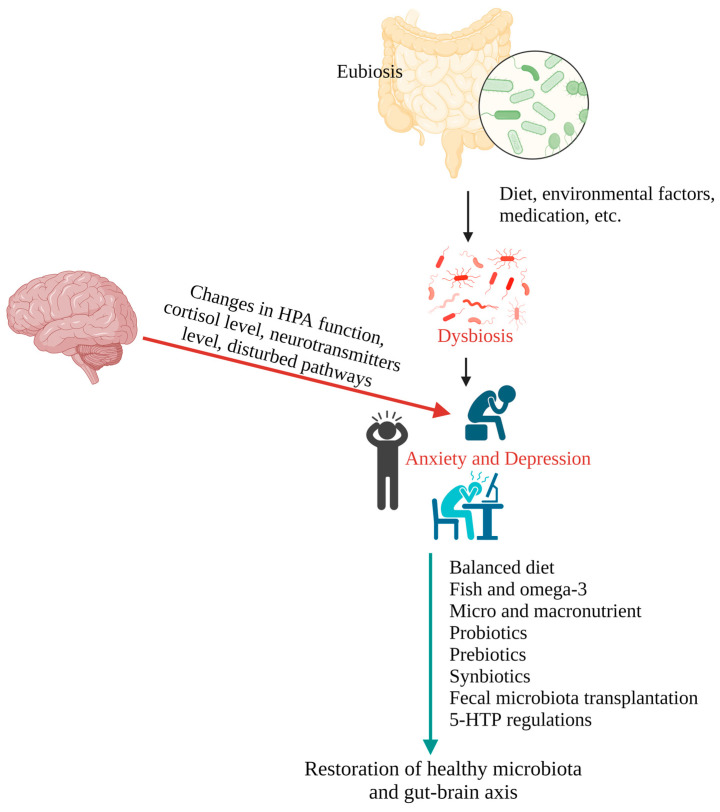
Illustration representing the factors associated with the development of anxiety and depression and the methods of gut microbiota restoration for the prevention and treatment of anxiety and depression.

Human depression may be treated with various synthesized drugs, though their effectiveness varies depending on several factors [180]. The recent alternative potential strategies currently being widely considered include modified diets, fish and omega-3 fatty acids intake, probiotics, prebiotics, synbiotics, postbiotics, fecal microbiota transplantation, and 5-hydroxytryptophan regulation (Table 2). These approaches directly or indirectly restore healthy gut microbial composition and diversity. Figure 2 represents the different methods of restoration of gut microbiota to prevent and treat anxiety and depression.

### 6.1. Modified Diet

Nutritional study has shifted away from concentrating on individual nutrients since they are hardly taken in the isolated form [197]. According to research, nutrient-dense food supports physical and mental health [138]. Proper brain function is supported by dietary nutrients such as vitamins, minerals, polyunsaturated fats, and amino acids in a balanced diet [140,141]. Numerous nutrients act as enzyme cofactors, producing neurotransmitters, cell signaling, and metabolic pathways [198]. Several different diets might aid in reducing anxiety and sadness. A decreased risk of anxiety or depression was linked to consuming a Mediterranean diet [199,200,201]. In individuals with low levels of anxiety and depression, this diet exhibits a preventive effect against unfavorable cardiovascular disease events [201]. The Mediterranean diet assures enough essential nutrients to prevent depression, such as fruits, nuts, vegetables, grains, legumes, and seafood. Intake of folate was negatively correlated with the frequency of depression in males, particularly smokers. Intake of the B12 vitamin was negatively correlated with depression in women, particularly in smokers and physically active women [202]. In addition, the Mediterranean diet alters the gut flora [203], which may be a plausible mechanism for reducing anxiety and depression. With an exposure–response connection, the “healthy Japanese” pattern may be inversely related to depressive symptoms. In addition to vegetables, fish/shellfish, and fruit, the “healthy Japanese” diet also featured potatoes, seaweed, mushrooms, and soy products. These seem to provide a dietary pattern less likely to cause inflammation. This aspect may be linked to improved psychological health via the production of monoamines and gut flora [204]. Even a fiber-rich diet reduces intestinal pH, preventing harmful bacteria from overgrowing [205]. Extensive research has shown the potential advantages of prebiotics, probiotics, and special dietary therapies in treating depression by modulating gut microbiota and depression through the gut–brain axis [206,207].

### 6.2. Fish and Omega-3 FA Intake

Healthy dietary habits that include fish have been linked to a decreased incidence of depression [22,208,209,210]. Both clinical and preclinical investigations have shown that fish oil is rich in omega-3 and exhibits antidepressant properties [211,212]. A meta-analysis of 13 randomized clinical trials found that fish oil demonstrated potential for treating serious depression [213]. A supplemental diet rich in omega-3 and omega-6 polyunsaturated fatty acids boosts *Bifidobacterium* and *Lactobacillus* and controls microbial metabolism, particularly during early life stress [212,214]. Omega-3 fatty acids, such as docosahexaenoic acid and eicosapentaenoic acid, have also been shown to improve cognition in adulthood and reduce stress and depression [212,215].

### 6.3. Micronutrient Intake

Many micronutrients are provided through the host’s diet and are also needed for the microbes. Therefore, the host’s micronutrient consumption may impact the composition and functioning of the intestinal flora. Mice receiving a diet low in magnesium had altered gut flora, which was linked to increased depression-like phenotypes [216]. Many bacteria need iron; therefore, iron consumption via diet impacts the diversity of the intestinal flora [217]. In addition, iron deficiency also has an impact on some neurotransmitter levels in the hippocampus and the corpus striatum [218]. People with depression are more iron-deficient than healthy individuals [219]. This result may explain the need for iron to produce neurotransmitters implicated in the pathophysiology of depression. Vitamin B alleviates anxiety, depression, and stress [173,174,220], and vitamin D3 alleviates depression [221,222,223,224].

### 6.4. Macronutrient Intake

A greater incidence of depression is substantially linked to a lower protein consumption than recommended. A 10% increase in protein consumption was shown to reduce the incidence of depression considerably in South Korea and in the United States [225]. Several biological explanations have linked the intake of protein and depression. These theories are supported by the fact that tryptophan, an amino acid, is a precursor of serotonin. Although consuming a large amount of protein may raise the plasma content of tryptophan [226], other neural amino acids can compete with tryptophan for brain cellular absorption [227]. As a result, increased protein consumption does not always result in higher levels of tryptophan in the brain. The finding that increased protein consumption protects against depression through boosting serotonin in the brain is challenging to understand because of this contradictory impact of protein intake on the tryptophan content. Additionally, other macronutrients have the power to control tryptophan levels and synthesis; for example, consuming carbs or receiving an insulin injection has been shown to raise plasma tryptophan levels [228]. Focusing on the impacts of macronutrients on the intestinal flora has led to an increasing convergence in nutrition [229]. Plant protein, unsaturated fats, and fiber encourage a healthy gut flora compared to excessive animal protein intake, saturated fats, and simple or artificial carbohydrates.

The quality of macronutrients, particularly dietary carbohydrates, is another factor to consider. The impact of high- and low-glycemic-load meals on depression symptoms was investigated in nondepressed persons. According to this research, eating a diet high in glycemic load may result in overall mood changes, more tiredness, and depression symptoms than eating a diet low in glycemic load [230]. The physiological effects of fatty acids vary depending on their type. However, no clinical trial data are available on how fatty acids affect depression or depressed symptoms depending on their saturation level. Since inflammation and endothelial dysfunction are significant risk factors for depression and cardiovascular disease, dietary advice for preventing cardiovascular disease may be beneficial for managing and preventing depression. The prevention and treatment of depression may be aided by replacing saturated fats with unsaturated fatty acids [231], although more research is required to confirm this statement.

### 6.5. Probiotics

Probiotics, living microorganisms, encourage the development of beneficial bacteria [54,232,233]. When the probiotic *L. rhamnosus* was administered to stressed mice, it decreased corticosterone levels and the stress-induced gamma-aminobutyric acid 2 mRNA expression. It did not affect gamma-aminobutyric acid 2 expression in the hippocampus [92,234]. Treatment with the probiotic *L. farciminis* reduced gut barrier leakiness [91]. In animal models, administering a probiotic such as *Bifidobacterium longum* restored hippocampus BDNF levels and decreased inflammation-induced anxiety-like behavior [183,234]. Probiotics have been observed to lessen depressive-like behavior in IBS patients, but not anxiety [235]. The *Oscillibacter* strain aids in treating insomnia and anxiety [236,237]. *B. longum* is beneficial in reducing stress-induced cortisol levels and daily self-reported stress levels [238]. Probiotics including *B. bifidum*, *B. lactis*, *Lactococcus lactis*, *L. casei*, *L. salivarius*, *L. brevis*, and *L. acidophilus* showed promising results in reducing negative thoughts and behavior in another study [239]. These results from studies demonstrate that probiotics may be used to treat depression and anxiety.

### 6.6. Prebiotics

Prebiotics are specific substrates that support the development and activity of certain advantageous gut microorganisms [240,241]. In healthy young volunteers, dietary prebiotics, including fructo-oligosaccharides (FOS) and galacto-oligosaccharides (GOS), encouraged the growth of advantageous bacteria such as *B. longum*. They decreased the hypothalamic–pituitary–adrenal axis activation caused by stress [206,242,243]. Rats’ anxiety and depressive-like behavior caused by lipopolysaccharides were decreased after receiving GOS [207]. Crocin-I enhanced gut microbiota composition and short-chain fatty acid levels and improved the brain-derived neurotrophic factor level in mice with depression-like phenotypes [244].

### 6.7. Synbiotics

Synbiotics may improve gut microbial activity. Malondialdehyde and hydrogen peroxide concentrations in human plasma were significantly reduced after taking syn-biotics [245]. Women consuming synbiotics had considerably greater plasma levels of glutathione and free sulfhydryl groups than males [246,247]. According to a randomized trial, synbiotic FOS, GOS, and inulin combined with a probiotic mixture containing *B. lactis*, *B. bifidum*, *L. acidophilus*, and *B. longum* reduced depression and increased serum levels of a brain-derived neurotrophic factor in depressed patients compared to controls. According to this study, synbiotics reduced depressive symptoms more than probiotics alone [181].

### 6.8. Postbiotics

Postbiotics have therapeutic effects similar to those of probiotics in that they support the integrity of the epithelial barrier function, restore the microbiota’s diversity and composition, control immunological responses, and regulate signaling along the gut–brain axis [248,249]. The administration of a heat-killed *L. helveticus* strain reduced anxiety- or depression-like phenotypes in adolescent male mice. It improved the genes involved in neuron differentiation and development and signal transduction in the nucleus accumbens [250]. Adult male mice were given heat-killed *Enterococcus fecalis* along with diet, which decreased depressive and anxious-like behaviors, increased the expression of the genes for the neurotransmitter receptors, and increased the density of *Butyricicoccus* and *Enterococcus* content in the gut [251]. Young individuals subjected to chronic stress were given two tablets of heat-inactivated *L. gasseri* daily for 24 weeks to lower their anxiety, improve their sleep, produce more n-valeric acid, and restore the balance of their microbiome [252].

### 6.9. Fecal Microbiota Transplantation (FMT)

FMT repairs gut diversity by transferring healthy microflora to the patient’s gut. FMT was developed to achieve healthy gut microbial composition and function, much like probiotics. When healthy donors’ fecal microbiota was transferred to anxious mice, it resulted in a reduction in the symptoms of anxiety and depression. FMT is now one of the approaches most often used to treat gastrointestinal and neuropsychiatric diseases [253]. IBS and other GI tract-related issues have been linked to depression in clinical trials. FMT from a healthy donor reduced IBS patients’ depressive and anxious-like behavior, and *Clostridium difficile* infection was also reduced in older patients [254,255]. However, if fecal microbiota is transferred from an unhealthy individual, it may cause adverse side effects such as depression. For example, according to a report, fecal microbiota transferred from rheumatoid arthritis patients with depression caused depression-like behaviors in mice via systemic inflammation [256]. Therefore, extra precautions are required for FMT procedures.

### 6.10. Bifidobacterium and 5-HTP Regulation

In a study, oral 5-HTP treatment markedly improved gut microbiota dysbiosis in mice exhibiting depression-like phenotypes. When 5-HTP was used to treat depression in rats, it helped maintain levels of short-chain fatty acids and brain-derived neurotrophic factors [257]. In a different study, mice underwent a 5-week trial of chronic moderate stress and received LAB (*B. longum* subsp. *infantis* E41 and *B. breve* M2CF22M7). E41 and M2CF22M7 dramatically decreased depression-like phenotypes in the mice by increasing Tph1 expression and 5-HTP secretion in RIN14B cells. 5-HTP and brain-derived neurotrophic factor concentrations in the brain were elevated after E41 and M2CF22M7 administration [258].

## 7. Advantages and Limitations of Drug Therapy and Gut Microbiota-Based Approaches

Tricyclic antidepressants (TCAs), antihistamines, SSRIs, serotonin-norepinephrine reuptake inhibitors (SNRIs), benzodiazepines (BZDs), and monoamine oxidase inhibitors (MAOI) are FDA-approved anti-depressants [259]. However, there are several drawbacks to popular anti-depressants, including drug tolerance, delayed action, inadequate effectiveness, and side effects [260]. Therefore, it is essential to find novel antidepressant approaches to guarantee the quality of life for all patients with depression and anxiety.

Microbial dysbiosis has a role in the pathophysiology of several chronic illnesses, including depression and anxiety. The above-stated therapies are based on microbiome restoration for treating and preventing depression and anxiety. However, procedures for the restoration of gut microbiota have several pros and cons, which are given below:

### 7.1. Advantages

- These therapies may correct certain forms of dysbiosis and promote health-promoting microbial loads.
- The effectiveness of the above-stated therapies has been proved by experiments using animal models and human subjects.
- The therapies are relatively effective and produce a long-term cure for the illness.

### 7.2. Limitations

- It is challenging to justify timing and dose regimens because conceptual, mechanistic, and ecological knowledge of the above-stated therapies is currently poor.
- Exposing patients’ immune systems to allogenic strains may be harmful if they suffer from disorders such as allergy disease, IBD, or autoimmune diseases characterized by pathologic immune responses.
- They are time-consuming procedures compared to drug therapies.

## 8. Conclusions and Future Prospects

Many studies have examined the gut microbiota in anxiety and depression disorders to elucidate underlying microbial relationships and guide potential diagnostic and therapeutic approaches for these issues. Worldwide, depression and anxiety are the most prevalent diseases and are associated with a reduction in patients’ quality of life. Epidemiology research has demonstrated the protective benefits of modified diets, fish and omega-3 fatty acid intake, probiotics, prebiotics, synbiotics, postbiotics, fecal microbiota transplantation, and 5-hydroxytryptophan regulation against anxiety and depression. In preclinical and clinical studies, the anti-depressive effects of these therapies occurred through various mechanisms, including the upregulation of neuroactive substance expression, the control of monoamine neurotransmitter levels, the reduction of oxidative stress and inflammation, and the modulation of the hypothalamic–pituitary–adrenal axis and gut–brain axis. Apart from the above-mentioned modalities, vagus nerve stimulation is one of the effective methods to attenuate anxiety and depression. Still, detailed studies are required to demonstrate its efficiency in improving mental health.

The available studies have some limitations. For example, the fundamental concepts underlying the processes by which the gut microbiome contributes to depression and anxiety and the efficacy of microbial restoration therapies need further understanding. Additionally, knowledge is poor regarding the effectiveness and safety of probiotics, prebiotics, synbiotics, postbiotics, and fecal microbiota transplantation. The most efficient dosages of fish and omega-3 fatty acids, probiotics, prebiotics, synbiotics, postbiotics, and fecal microbiota transplantation, as well as the possible function of adjuncts such as antidepressant drugs, have not been determined.

Further studies are needed to assess the effectiveness of microbial restoration therapy in anxiety and depression among relevant patient groups. Furthermore, adequately powered clinical and follow-up studies are required to determine the response stability and short- and long-term safety. In addition, basic research is required to understand the mechanisms behind microbiota-based treatments for mental illness, especially for anxiety and depression.

## Figures and Tables

**Table 1:** The microbiota changes in depression and anxiety.

Disease	Subject	Sample Size and Characteristics	Sequencing Platforms	Observations and Changes in Microbiota	Ref.
MDD (with anxiety)	Human	Control: *n* = 10 (Mean age: 33 years, 60% female); Psychiatric subjects: *n* = 60 (major depressive disorder and anxiety: *n* = 38, anxiety: *n* = 8, depression: *n* = 14).	16S rRNA sequencing using Roche 454 Titanium platform.	↓ *Clostridia* in people with depression. ↓ *Bacteroides* are more closely linked to the prevalence of anxiety than depression.	[90]
Depression	Flinders sensitive line rats	24 FRL rats (weight 327.8 ± 40.7 g, and 10.6 ± 1.1 weeks old).	16S rRNA sequencing using Illumina MiSeq.	↓ Phyla *Elusimicrobia* and *Saccharibacteria* ↑ Proteobacteria ↑ *Blautia* and *Subdoligranulum* ↓ *Candidatus Saccharimonas*, *Alistipes*, and *Roseburia*	[145]
Depression	Human	Belgian Flemish Gut Flora Project population (*n* = 1054 subjects).	Shotgun sequencing	In depression, ↓ *Coprococcus* and *Dialister* after restricting the antidepressant medication effect. ↓ *Fusicatenibacter* and *Butyricicoccus* after controlling for antidepressant treatment. ↑ *Phascolarctobacterium*, *Lactobacillus*, *Parabacteroides*, *Holdemania* ↓ *Dialister*, *Coprococcus*, *Turicibacter*, and *Faecalibacterium*	[148]
Bipolar depression	Human	Healthy controls: *n* = 45; Bipolar depression patients: *n* = 72.	16S rRNA sequencing using Illumina MiSeq.	↑ *Parabacteroides*, *Bacteroides*, *Weissella*, and *Halomonas*.	[149]
MDD	Human	Healthy controls: *n* = 71; Major depressive disorder patients *n* = 70.	16S rRNA sequencing using Roche 454 Titanium platform.	↓ *Bacteroidetes*, *Actinobacteria*, and *Firmicutes*	[150]
Anxiety and depression	Human	Control: *n* = 46; Anxiety and depression group: *n* = 23.	16S rRNA sequencing using MiniSeq.	Reduction in *Gemmiger*, *Ruminococcus*, and *Veillonella*.	[151]
Generalized anxiety disorder	Human	Healthy controls: *n* = 36; Generalized anxiety disorder patients: *n* = 40.	16S rRNA sequencing using Illumina MiSeq.	↑ *Fusobacterium*, Escherichia-Shigella, and *Ruminococcus gnavus*	[152]
Chronic paradoxical sleep deprivation-induced depression	Wistar rats	-	16S rRNA pyrosequencing.	↓ *Akkermansia, Phascolarctobacterium,* and *Ruminococcus* ↑ *Parabacteroides, Oscillospira,* and *Aggregatibacter*	[153]
MDD	Human	Healthy controls: *n* = 10 (age: 24–65 years, women: *n* = 5); Major depressive disorder patients: *n* = 10 (age: 18–56 years, women: *n* = 5).	-	↑Actinobacteria, Firmicutes, and Lachnospiraceae ↓ Bacteroidetes and Proteobacteria ↓ *Faecalibacterium*	[154]
Late-life depression	Human	Healthy controls: *n* = 17; Late-life depression patients: *n* = 36.	16S rRNA sequencing using Illumina MiSeq.	↑ Akkermansiaceae and *Akkermansia*.	[155]
MDD	Human	Healthy controls: *n* = 37; Major depressive disorder patients: *n* = 36.	16S rRNA sequencing using Illumina MiSeq.	↑ Actinobacteria and Firmicutes ↑ *Bifidobacterium*, and *Blautia* ↓ *Prevotella*	[156]
MDD	Human	Healthy controls: *n* = 10; Major depressive disorder patients: *n* = 10.	16S rRNA sequencing using Illumina MiSeq.	↓ *Bifidobacterium* and *Dialister* ↑ Bacteroidetes and *Bacteroides*	[157]
Depression	Human	Controls: *n* = 31; Ulcerative colitis without depression: *n* = 31; Ulcerative colitis with depression: *n* = 31.	16S rRNA pyrosequencing.	↑ Proteobacteria, gamma proteobacteria ↓ Firmicutes, Clostridia, and Clostridiales	[158]
Postpartum depressive disorder	Human	Healthy controls: *n* = 16; Postpartum depressive disorder patients: *n* = 28.	16S rRNA sequencing using Illumina MiSeq.	↓ Faecalibacterium, Phascolarctobacterium, Butyricicoccus, and Lachnospiraceae ↑ Enterobacteriaceae	[159]
Systemic lupus erythematosus with depression	Human	Healthy controls: *n* = 32; Systemic lupus erythematosus with depression patients: *n* = 21.	16S rRNA using Illumina Novaseq 6000 sequencing.	↓ Ratios of the genera *Faecalibacterium* to *Roseburia* and phyla Firmicutes to Bacteroidetes.	[160]
MDD	Human	Healthy controls: *n* = 43; First-episode, drug-naïve major depressive disorder patients: *n* = 66.	16S rRNA sequencing using Illumina Novaseq PE250 platform.	↑ Deinococcus and Odoribacter ↓ *Bacteroides*, *Alistipes*, *Turicibacter*, *Clostridium*, *Roseburia*, and *Enterobacter*	[161]
MDD	Human	Healthy controls: *n* = 28; Major depressive disorder patients: *n* = 26.	16S rRNA sequencing using Illumina HiSeq 2500 platform.	↓ Firmicutes ↑ Proteobacteria and Actinobacteria.	[162]
MDD	Human	Healthy controls: *n* = 45; Current active major depressive disorder patients: *n* = 46; Remission or with only mild symptoms of major depressive disorder: *n* = 22.	16S rRNA sequencing using Illumina MiSeq.	↑ *Bilophila* and *Alistipes* ↓ *Anaerostipes* and *Dialister*	[163]
MDD	Human	Healthy controls: *n* = 27; Major depressive disorder patients: *n* = 27.	16S rRNA sequencing using Illumina HiSeq2500.	↓ *Lachnospiraceae*, *Ruminococcaceae*, *Coprococcus*, *Blautia*, *Clostridiaceae*, and *Dorea* ↑ *Oxalobacter*, *Pseudomonas*, *Parvimonas*, *Bulleidia*, *Peptostreptococcus*, and *Gemella*	[164]
MDD	Human	Healthy female controls: *n* = 24; First-episode drug-naïve major depressive disorder female patients: *n* = 24; Healthy male controls: *n* = 20; First-episode drug-naive major depressive disorder male patients: *n* = 20.	16S rRNA sequences using Roche 454.	↓ *Bacteroidetes* and *Proteobacteria* ↑ *Firmicutes* and *Actinobacteria*	[165]
MDD	Human	Healthy controls: *n* = 29; Major depressive disorder patients: *n* = 26.	16S rRNA sequencing using Illumina HiSeq 2500 platform.	↑ *Bifidobacterium*, *Enterococcus*, *Megasphaera*, *Coriobacterium*, *Streptococcus*, *Slackia, Heliobacterium*, *Lactobacillus*, *Oscillibacter*, *Olsenella*, *Sphaerochaeta*, *Desulfitobacterium*, *Acidaminococcus*, *Eggerthella*, *Lachnoclostridium*, *Atopobium*, *Rothia.* ↓ *Sphingobacterium*, *Bacteroides.* ↑ *Clostridium saccharolyticum*, *Megasphaera elsdenii*, *Acidaminococcus fermentans*, *Streptococcus parasanguinis*, *Eggerthella lenta*, *Desulfovibrio vulgaris*, *Lactobacillus crispatus*, *Bifidobacterium adolescentis*, *Enterococcus faecium*, *B. longum*, *Atopobium parvulum*, *B. bifidum.* ↓ *Bacteroides helcogenes.*	[166]
MDD	Human	Healthy controls: *n* = 30; Major depressive disorder patients: *n* = 31; Bipolar disorder with current major depressive episode patients: *n* = 30.	16S rRNA sequencing using Illumina HiSeq 2500 platform.	↓ *Firmicutes*, *Bacteroidota* ↑Actinobacteria ↑ *Bacteroides*, *Clostridium*, *Bifidobacterium*, *Oscillibacter*, and *Streptococcus*	[167]

MDD: Major depressive disorder; ↑: Increased; ↓: Decreased.

**Table 2:** Supplements that aid in the management of depression and anxiety.

Intervention	Subjects	Main Results	Ref.
*L. rhamnosus*	Mice	Lowered stress-induced anxiety and depressive-like behavior	[92]
*Lactobacillus acidophilus*, *B. longum*, *B. lactis*, *B. bifidum*, galacto-oligosaccharides (GOS), inulin and fructo-oligosaccharides (FOS)	Human	The synbiotic intervention reduced the depression and anxiety symptoms effectively	[181]
*Bacillus coagulans*	Human	Reduction of depression and irritable bowel syndrome symptoms	[182]
*Bifidobacterium longum*	Human	Improved the quality of life and lessened depression, but not anxiety, in patients with irritable bowel syndrome.	[183]
*Clostridium butyricum*	Human	Enhanced the effectiveness of traditional treatment for depression	[184]
*L. acidophilus*, *L. casei*, and *B. bifidum*	Human	Reduction of Beck’s Depression Inventory score	[185]
*L. helveticus* R0052 and *B. longum*	Human	Effectively reduced symptoms of depression and decreased anxiety	[186]
*L. helveticus* and *B. longum*	Human	Improvement of Beck’s Depression Inventory score	[187,188]
*L. plantarum*	Human	Improvement in symptoms and psychological scores	[189]
*B. longum* subsp. *longum* BAMA-B05/Bau-B1024, *B. lactis* BAMA-B06/Bau-B0111, *B. adolescentis*, *Streptococcus thermophiles*, *L. acidophilus*, and *L. delbrueckii* subsp. *bulgaricus*	Human	Decreased anxiety.	[190]
*S. thermophiles*, *B. longum*, *B. breve, L. rhamnosus*, *L. bulgaricus*, *L. acidophilus*, *L. casei*, and FOS	Human	Improved depressive symptoms in major depressive disorder	[191]
Short-chain FOS	Human	Improved depression and anxiety score in patients with irritable bowel syndrome	[192]
FOS and GOS	Mice	Improved depression and anxiety	[193]
Fecal microbiota transplantation	Mice	Reduced stress-associated depressive-like behavior	[194]
Oligosaccharides 3′sialyllactose (3′SL) or 6′sialyllactose (6′SL)	Mice	Lower nervous anxiety-related reactions and preventative impact on anxious behavior.	[195]
Polysaccharide of okra	Mice	Restored the gut microbiota	[196]

## Data Availability

Not applicable.

## Abbreviations

IBD	Inflammatory bowel disease
CRP	C-reactive protein
4EPS	4-ethyl phenyl sulfate
BBB	Blood–brain barrier
GABA	Gamma-aminobutyric acid
IL	Interleukin
HPA	Hypothalamic–pituitary–adrenal
DNA	Deoxyribonucleic acid
RNA	Ribonucleic acid
BDNF	Brain-derived neurotrophic factor
5-HTP	5-hydroxytryptamine
SDV	Subdiaphragmatic vagotomy
LPS	Lipopolysaccharides
MDD	Major depressive disorder
SSRIs	Selective serotonin reuptake inhibitors
FRL	Flinders resistant line
GI	Gastrointestinal
IBS	Irritable bowel syndrome
FA	Fatty acids
TCAs	Tricyclic antidepressants
SNRIs	Serotonin-norepinephrine reuptake inhibitors
BZDs	Benzodiazepines
MAOI	Monoamine oxidase inhibitors
FDA	Food and Drug Administration
FMT	Fecal microbiota transplantation
FOS	Fructo-oligosaccharides
GOS	Galacto-oligosaccharides
mRNA	Messenger ribonucleic acid
